# Bioavailability Study of Maqui Berry Extract in Healthy Subjects

**DOI:** 10.3390/nu10111720

**Published:** 2018-11-09

**Authors:** Christiane Schön, Roland Wacker, Antje Micka, Jasmin Steudle, Stefanie Lang, Bernd Bonnländer

**Affiliations:** 1 BioTeSys GmbH, Schelztorstr. 54–56, 73728 Esslingen, Germany; c.schoen@biotesys.de (C.S.); r.wacker@biotesys.de (R.W.); a.micka@biotesys.de (A.M.); j.steudle@biotesys.de (J.S.); 2 Anklam Extrakt GmbH, Marienbergstr. 92, 90411 Nuremberg, Germany; stefanie.lang@anklam-extrakt.de

**Keywords:** maqui berry extract, anthocyanins, human, delphinidin, bioavailability, anti-oxidative

## Abstract

Several health promoting effects have been reported for maqui berry, rich in anthocyanins. Direct effects of anthocyanins as well as bioactive metabolites might be involved. Within the study, bioavailability of a proprietary standardized maqui berry extract Delphinol^®^ was investigated based on two selected anthocyanins (delphinidin-3-*O*-glucoside (DS) + cyanidin-3-*O*-sambubioside (CS)) and two breakdown products (protocatechuic acid (PCA) + gallic acid (GA)) after a single-dose supplementation in humans. Pharmacokinetic parameters were calculated from individual concentration time curves. In all 12 subjects a significant increase was noted in plasma values of DG and CS after intake of maqui berry extract. Maximum concentration of DG was observed after 1.0 ± 0.3 h and CS after 2.0 ± 1.1 h. Within 8 h, concentrations nearly returned to baseline levels. The results confirm a fast uptake and metabolism of the two selected key substances. Additionally, the phenolic acids GA and PCA were observed as breakdown products of anthocyanins. In summary, the study clearly confirms the bioavailability of maqui berry extract and its specific anthocyanin compounds and related breakdown products in healthy subjects.

## 1. Introduction

Fruits and vegetables are rich in polyphenols and flavonoids. Research in the field has indeed provided evidence of the anti-oxidative and anti-inflammatory activities of polyphenols and their health-promoting benefits [1], which is also supported by the World Health Organization [2]. The association of high fruit and vegetable intake was extensively examined in individuals with non-communicable diseases in western countries such as coronary heart disease, stroke, obesity and type 2 diabetes. In this context anthocyanins, which are water-soluble polyphenolic phytochemicals found in high amounts in red and purple coloured fruits, are considered to be strong antioxidants and have been shown to impact overall health [3].

Maqui berries (*Aristotelia chilensis*), purple-black berries domestic in Chile and Argentina, contain a rich variety of anthocyanins [4]. While all anthocyanins have an antioxidant activity, the delphinidins represent the most potent anti-oxidative group. The richest natural source of delphinidin is the maqui berry from which a standardized extract is available [5]. Several health benefits for this specific maqui berry extract (MBE) have been previously demonstrated for example in blood glucose lowering effects, in increasing tear fluid production or in anti-oxidative effects in general [6,7,8,9]. Furthermore, pure delphinidin-3-*O*-glucoside also predominantly present in MBE was able to decrease platelet activity and therefore may contribute to an improvement in blood circulation [10].

Although the bioavailability of anthocyanins is limited, estimates are of about 1% [11,12]. Different causes may contribute to the apparent low bioavailability, for example, cellular uptake in the intestine, first pass metabolism, low absorption rate and limited stability during pass through the intestinal tract [11,13].

In any case, health-promoting benefits are discussed based on a different mode of action. Besides the interaction with gut microbiota, direct effects of anthocyanin structures or metabolites are additionally described [14,15]. It is becoming clearly recognized that anthocyanins can interact with various molecular targets and affect multiple signalling pathways [16,17,18,19,20,21]. To further evaluate the clinical benefits observed with the MBE [6,7,8,9] it is of high interest to specifically examine the uptake of unmetabolized anthocyanin structures present in the extract. Two anthocyanins (delphinidin-3-*O*-glucoside (DS) + cyanidin-3-*O*-sambubioside (CS)), part of the proprietary MBE were selected to characterize the bioavailability. Furthermore, human blood samples were screened for metabolites (protocatechuic acid (PCA) + gallic acid (GA)) that are known breakdown products of anthocyanins.

## 2. Study Design and Methods

### 2.1. Participants

This study was conducted according to the guidelines of the Declaration of Helsinki and Good Clinical Practice. The protocol and all documents were approved by the Institutional Review Board (IRB) of Landesärztekammer Baden-Württemberg with the reference number F-2017-083. The study was registered in www.clinicaltrial.gov (NCT03485885). 

Subjects were recruited through the subject database of BioTeSys GmbH (Esslingen am Neckar, Germany). Non-smoking female and male participants aged 18–50 years with a BMI ≥ 19 or ≤30 kg/m^2^ were screened to ascertain their eligibility to participate in the study. Subjects had to be in good physical and mental health as established by the medical history, physical examination, electrocardiogram, vital signs, results of biochemistry and haematology and be willing to sign the Informed Consent Form prior to screening evaluations. The main exclusion criteria were a relevant history or presence of any medical disorder, potentially interfering with this study (e.g., mal absorption, chronic gastro-intestinal diseases, etc.), high coffee consumption (>3 cups per day), high fruit and vegetable consumption (>5 servings per day), regular intake of drugs or supplements possibly interfering with this study (e.g., Vitamin C, E, oligomeric proanthocyanidins (OPC), etc.), drug-, alcohol- and medication abuses, relevant allergy or known hypersensitivity against compounds of the study preparations.

### 2.2. Study Design

The study was performed as a monocentric, single-armed study with 12 eligible subjects at the study centre of BioTeSys GmbH, Esslingen, Germany. During screening, nutrition and life style habits were reported and possible confounding factors were checked. Three days prior to visit 1, subjects were asked to limit their consumption of foods rich in polyphenols and additionally 30 h prior to the kinetic day abstain from coffee, black tea and alcohol. Furthermore, subjects consumed a standardized dinner (bread with cream cheese) the evening prior to visit 1. No strenuous physical activity or endurance sports were allowed within 24 h before the study visit. Medications for the treatment of chronic diseases that do not affect the metabolism of the study product were permitted and judged individually by the investigator, regarding their interference with the study. Any concomitant medication used chronically or for the treatment of adverse events (AEs) was documented.

At visit 1, subjects received a single dose of MBE in fasting conditions after insertion of a permanent venous catheter and baseline blood sampling. Blood was further sampled at 0.5, 1, 1.5, 2, 3, 4, 6 and 8 h after the single dose intake, to analyse DG, CS, GA and PCA. Fluid intake and meals were standardized during the kinetic day, meals were served at 2 h (butter pretzel) and 5 h (spaghetti carbonara) after intake of the study product.

### 2.3. Intervention

*Aristotelia chilensis* standardized berry extract was provided by Anklam Extrakt GmbH (Anklam, Germany). The proprietary, standardized extract is produced under GMP conditions and marketed under the trade names Delphinol^®^, MaquiBright^®^, maquisupreme^®^, BrightSight^®^. The appearance of the extract is a purple powder, provided in hard gelatin capsules of 250 mg of extract per capsule. The extract is standardized to ≥35% of total anthocyanins and ≥25% delphinidins. A typical HPLC profile of the extract is attached in Supplementary Information, Figure S1. Safety assessments for the extract are available, clearly demonstrating the safe use of MBE in vitro and in vivo.

Four capsules (1000 mg Delphinol^®^) were administered in a single dose. The capsules were taken under fasting conditions after the baseline blood sampling with 200 mL of still water. The study personnel administered the study product and intake was under supervision.

### 2.4. Sample Collection and Processing

At screening, venous blood was sampled to determine safety parameters (differentiated hemogram and clinical laboratory (liver enzymes (alanine aminotransferase, aspartate aminotransferase, γ-glutamyl transferase, alkaline phosphatase), lipid profile (total cholesterol, low density lipoprotein- and high density lipoprotein-cholesterol, triglycerides), creatinine, uric acid and fasting glucose). Analysis was performed the same day in an accredited lab Synlab Medizinisches Versorgungszentrum Leinfelden-Echterdingen, Germany with standard methods.

For biomarker analysis, venous blood samples were collected into 7.5 mL Ethylenediaminetetraacetic acid- monovettes (Sarstedt) and samples processed under light-protected conditions. Plasma was separated shortly after sampling by centrifugation at 3000× *g* for 10 min at 4 °C. Plasma aliquots were stabilized with formic acid 50% for anthocyanin analyses. Processing time was below 30 min until freezing (<−20 °C) of plasma aliquots. 

In a pre-test, stability of spiked plasma samples was checked under study conditions. The relevant analytes are recognized to be stable at 4 °C for at least 45 min. Therefore, no impact of pre-analytics is expected in the study samples. 

### 2.5. Methods for Safety (Adverse Events, Concomitant and Tolerability)

Between screening and visit 1, volunteers had to document any adverse event and concomitant medication in the volunteer diaries. Tolerability was assessed after the kinetic day. Subjects had to determine tolerability according to three categories from “well tolerated”, “slightly uncomfortable” or “very uncomfortable”.

### 2.6. Methods for Assessing Efficacy Variables–Analysis of DG, CS, GA and PCA

Within the pre-test, key parameters were scanned to describe the bioavailability of MBE. With respect to the availability of the standard material and chromatographic conditions, DG and CS were selected as representatives of the anthocyanins present in the proprietary MBE. In addition, GA and PCA were selected for analysis since both are metabolites of anthocyanins respectively. 

A Waters OASIS HLB C18 SPE column for solid phase extraction (SPE) of plasma samples was conditioned with 1 mL methanol, equilibrated with 1 mL 1.5 M formic acid and 1 mL of plasma loaded onto the column. After a washing step with 1 mL 1.5 M formic acid followed by 1 mL 5% methanol, analytes were eluted with 0.1% formic acid in methanol and the eluate dried with a nitrogen stream at room temperature. The dry residue is reconstituted with 100 μL 5% formic acid in water and transferred to micro glass vials.

For calibration DG and CS standards (PhytoLab GmbH & Co. KG, Vestenbergsgreuth, Germany; Delphinidin-3-*O*-glucoside chloride (DG), PhytoLab 89627, CoA 89% (pure cation); Cyanidin 3-*O*-sambubioside chloride (CS), PhytoLab 89617, CoA 94% (pure cation)) were solved in 0.01% HCl in methanol, diluted in 5% formic and added to a basal plasma pool. Samples and standards were analysed with a Waters Acquity H-Class UPLC (Ultra-Performance Liquid Chromatography) equipped with an Acquity DAD and a Xevo-TQS micro mass spectrometer. Separation was achieved using a gradient separation with an Acquity UPLC BEH C18 column (1.7 μm, 2.1 × 50 mm). The gradient was composed of 5% formic acid in water and acetonitrile at a flow rate 0.7 mL/min. The gradient UPLC separation is summarized in Supplementary Material Table S1. The samples were kept at 10 °C, the column temperature was 30 °C and injection volume was 5 μL. UV-DAD data were collected between 190 and 800 nm wavelengths to monitor possible contaminations of samples and for general quality assurance purposes. The quantification of analytes took place by liquid chromatography–mass spectrometry (LC-MS/MS) in electrospray ionisation (ESI) multiple reaction monitoring (MRM) mode only as indicated in Supplementary Material Table S2. 

An internal standard 2-chloro-5-nitrobenzoic acid (MW 201, 56 g/mol, ClC_6_H_3_(NO_2_)CO_2_H, Sigma-Aldrich Art. No. 125113, Batch No. BCB1575) was added to plasma samples. However, the precision of the internal standards (IS) was rather low with relative standard deviation (RSD) 12.4% (*N* = 11), indicating instability and a peak deformation. The internal standard was selected based on the description in the literature [22]. As a result, under the very acidic conditions, which is necessary for anthocyanin stability, it was not appropriate for the intended purpose. Therefore, samples were quantified without consideration of the internal standard. The method characteristics were acceptable in regard to the low concentration and the instability of the analytes (precision: RSD < 13% (DG), RSD < 7% (CS) (standard addition to plasma), interassay precision 10.4% (DG) and 4.9% (CS). Limit of detection (LoD): 0.316 nmol/L (DG) (S/N ratio), 0.043 nmol/L (CS) (S/N ratio); Limit of quantification (LoQ): 0.451 nmol/L (DG) (S/N ratio), 0.060 nmol/L (CS) (S/N ratio); Recovery: 90% (DG)/84% (CS) (standard addition in matrix)). An example of multiple reaction monitoring (MRM) of plasma with spiked standards is attached in Supplementary Material Figure S2. 

Within the pre-test, a methodology was set up to selectively determine PCA and GA. The determination was accomplished according to Pang et al. [23]. The method is based on UHPLC-MS/MS detection using MRM in negative ESI mode.

Some additional testing indicated that concentrations of GA and PCA were below limit of quantification. Hence, it was decided to qualitatively assess GA and PCA within the method set up focusing on DG and CS and described above to depict data for GA and PCA only as areas.

An example MRM for all four analytes is presented in Supplementary Material Figure S3. 

### 2.7. Analysis Software and Statistical Analysis

All 12 subjects were included in the analysis. Statistical tests were performed two-sided and *p* values < 0.05 were considered significant. 

Non-normality was evaluated with the Shapiro-Wilk test with a significance level of 0.10. Efficacy parameters were evaluated with exploratory analyses.

For efficacy parameters, plasma concentration increase over time was analysed using ANOVA with repeated measures or Friedman test in case of non-normality. Pair wise tests were performed against baseline levels with an appropriate post-test (Dunnett’s Multiple Comparison test or Dunn’s Multiple Comparison test).

Pharmacokinetic parameters were individually calculated with the blood concentrations time curves: AUC_0–8 h_: Area under the observed concentration-time curve above baseline, C_max_: Observed maximum serum concentration. T_max_: Observed time to reach peak serum concentration C_max_.

Data are presented as mean and 95% confidence interval (CI). Statistical analysis was carried out using the software GraphPad Prism Version 5.04 (GraphPad Software, La Jolla, CA, USA).

## 3. Results

### 3.1. Participant Characteristics

**Figure 1:**
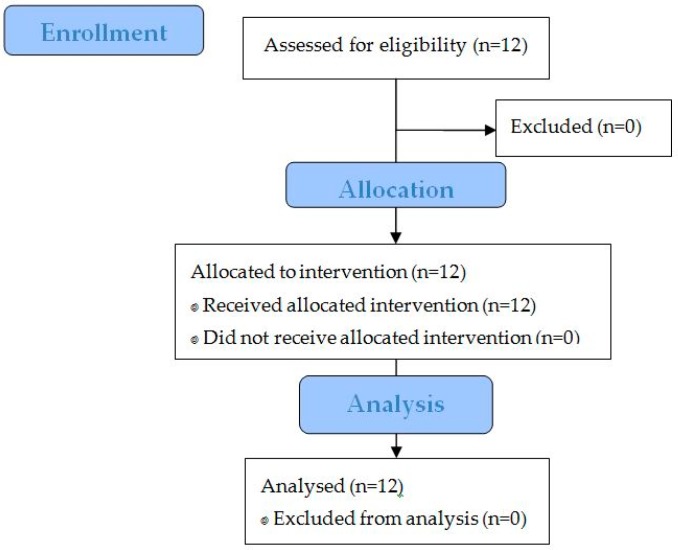
Flow diagram demonstrating patient recruitment

In November 2017 a total of 12 subjects were recruited via BioTeSys’ volunteer database and included, as shown in Figure 1. Overall, 22 subjects were pre-screened for eligibility and of those 12 invited for the screening visit. All 12 screened volunteers (6 women and 6 men) were included and completed the study successfully without considerable protocol deviations. The study terminated in December 2017. 

### 3.2. Study Performance

Investigated study population was on average 29.5 years (95% CI: 23.4–35.7) old. Male study participants had an average age of 28.2 years (95% CI: 19.1–37.3) and female volunteers had an average age of 30.8 years (95% CI: 19.0–42.7). Participants’ BMI was on average 23.6 kg/m^2^ (95% CI: 21.6–25.7) which is classified as normal. Table 1 shows the demographic data of the volunteers. On average, vital signs and blood routine parameters were within normal range. As inclusion criterion, the consumption of fruits and vegetables was limited to a maximum of 5 portions in sum. Consequently, no subjects with vegetarian or vegan diet styles were allowed to take part. Additionally, the majority of volunteers (83.3%) practiced sports regularly. Besides the use of an oral contraceptive (3 women), none of the subjects took any chronic medication. Overall, the study population was a healthy, non-smoking study group.

### 3.3. Analysis of Delphinidin-3-O-glucoside (DG) and Cyanidin-3-O-sambubioside (CS)

**Figure 2:**
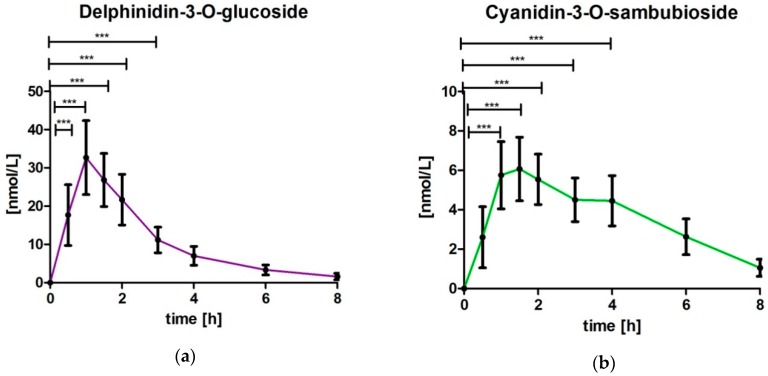
Delphinidin-3-*O*-glucoside and cyanidin-3-*O*-sambubioside concentration time profile after intake of maqui berry extract depicted as a summary curve of mean values at single time points (mean ± 95% CI, *** *p* ≤ 0.001): (**a**) delphinidin-3-*O*-glucoside (**b**) cyanidin-3-*O*-sambubioside.

DG and CS were not detected in baseline samples. In all subjects an increase was observed in the plasma values of DG and CS after intake of MBE (Figure 2). The plasma concentration of DG was significantly increased for the time points 0.5 h (*p* < 0.0001), 1 h (*p* < 0.0001), 1.5 h (*p* < 0.0001), 2 h (*p* < 0.0001) and 3 h (*p* < 0.01) compared to baseline levels. After 8 h the DG concentration nearly returned to baseline levels. The plasma concentration of CS was significantly increased for the time points 1 h (*p* < 0.0001), 1.5 h (*p* < 0.0001), 2 h (*p* < 0.0001), 3 h (*p* < 0.0001) and 4 h (*p* < 0.0001) compared to baseline levels and returned almost to baseline after 8 h.

### 3.4. Pharmacokinetic Parameters (AUC_0–8 h_, T_max_ und C_max_) for DG and CS

**Figure 3:**
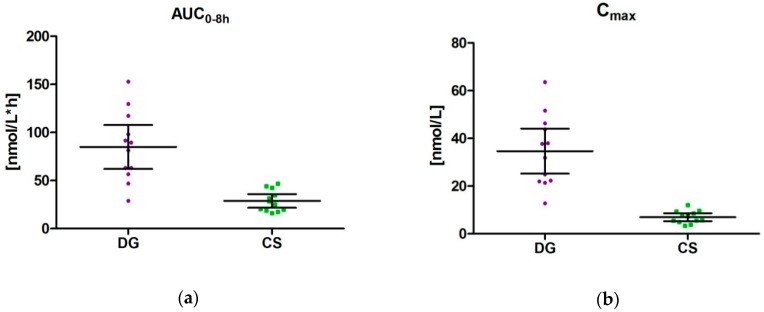
Scatter chart with pharmacokinetic endpoints calculated from individual concentration time curves after intake of maqui berry extract; mean ± 95% CI: (**a**) AUC_0–8 h_ [nmol/L*h] (**b**) C_max_ [nmol/L]; DG: delphinidin-3-*O*-glucoside; CS: cyanidin-3-*O*-sambubioside.

The AUC_0–8 h_ was 84.9 nmol/L*h (95% CI: 62.1–107.7) for DG and 28.9 nmol/L*h (95% CI: 21.8–35.9) for CS, resulting in a ratio between DG/CS of 2.94 (Figure 3). This represents the higher amount of DG in the study product. There was an inter-individual difference of AUC. The coefficient of variation was 42.4% for DG and 38.5% for CS.

C_max_ levels varied between 21.39–63.55 nmol/L for DG (mean: 34.7 (95% CI: 25.2–44.1)) and between 3.46–12.09 nmol/L for CS (mean: 7.0 (95% CI: 5.3–8.6)) resulting in factor 4.96 for DG. The coefficient of variation was 42.9% for DG and 37.5% for CS. 

The maximum concentration was observed for DG between 0.5 h and 1.5 h (mean: 1.0 h (95% CI: 0.81–1.19) and for CS between 1 h and 4 h (mean: 2.0 h (95% CI: 1.30–2.70)). 

### 3.5. Analysis of Gallic Acid (GA) and Protocatechuic Acid (PCA) 

**Figure 4:**
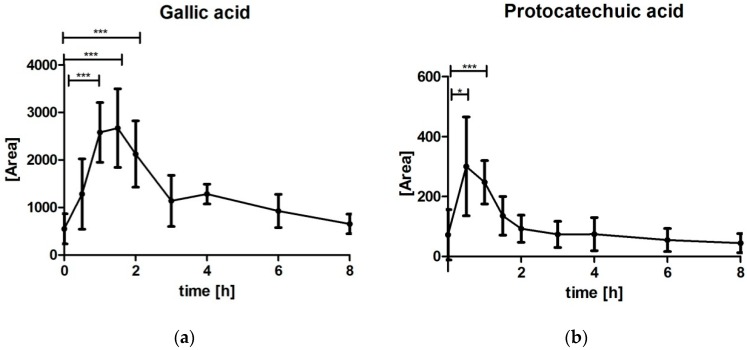
Gallic acid and protocatechuic acid concentration time profiles after intake of maqui berry extract presented as summary curves of mean values of area units at single time points (mean ± 95% CI, * *p* < 0.05; *** *p* ≤ 0.001): (**a**) Gallic acid [area units] (*N* = 8 due to exclusion of outliers) (**b**) Protocatechuic acid [area units] (*N* = 12).

Both breakdown products GA and PCA were below limit of quantification (DG: <15.87 nmol/L; PCA: <284.19 nmol/L) (Figure 4). Despite the limited precision and sensitivity to confounding factors due to signal noise ratio, data are presented as areas to illustrate some preliminary effect of metabolism. Data clearly described an increase of GA and PCA after intake of MBE. Maximum area of PCA and GA was identified for most subjects at 0.5 h to 1.5 h time point indicating a very fast metabolism.

### 3.6. Safety Assessments

Overall, tolerability of the study preparation was satisfactory. All volunteers (100%) mentioned that study preparation was well tolerated during the kinetic day. No adverse event was reported during the kinetic day. 

## 4. Discussion

### 4.1. Delphinidin-3-O-glucoside (DG) and Cyanidin-3-O-sambubioside (CS)

Bioavailability of bioactive ingredients is the precondition for any systemic effect, which is also valid for anthocyanins [24]. Based on literature, a unique pattern is identified compared to other flavonoids after oral administration of anthocyanins [25]. For the proprietary *Aristotelia chilensis* berry extract, the uptake pattern of anthocyanins was determined for two selected key substances.

Assessing the bioavailability of the key parameters DG and CS present in the MBE, after intake of the extract, revealed a very fast and significant increase of DG and CS in its native form without metabolizing. The uptake pattern indicated one kinetic peak for DG, most subjects reaching the maximum concentration after 1 h (T_max_: 1.0 ± 0.3 h; range: 0.5–1.5 h). For CS the maximum concentration was reached slightly but significantly later with T_max_: 2.0 ± 1.1 h; range: 1–4 h (*p* = 0.0131 in comparison to DG). Furthermore, for CS a minor pronounced second peak or slight delay was observed between 3 and 4 h. It is possible that the two peaks appeared in relation to the time at which the anthocyanins were absorbed. For anthocyanins different resorption sites are considered, for example, oral cavity, stomach and jejunum [11]. Due to the encapsulation, the buccal uptake can be excluded, whereas uptake from stomach and jejunum is expected. The observed T_max_ values for these selected anthocyanins are in line with reported data in the literature [25]. 

The higher C_max_ levels of DG measured in blood in comparison to CS reflect the higher content of DG in the study product, however does not reflect the same ratio as present in the product (product: DG: 7.77% equivalent to 167 µmol, CS: 0.97%, equivalent to 16.7 µmol; ratio DG/CS: 10.0; C_max_ level DG/CS: 4.96). According to the literature, the uptake from stomach and jejunum might also depend on the chemical structure. Talavera et al. [26] reported in an animal study with rats that after in situ perfusion, anthocyanins were absorbed efficiently by the jejunum and ileum. The absorption was influenced by the chemical structure of the anthocyanins and ranged from 10.7% (malividin-3-*O*-glucoside) to 22.4% for cyanidin-3-*O*-glucoside. Additionally, the metabolism in the gastrointestinal tract might also differ among chemical structures of the anthocyanins, contributing to the shift between relation of the selected anthocyanins in the product and the occurrence in plasma.

Once entering the circulation, extensive and fast first-pass metabolism occurs. De Ferrars et al. [27] reported 17 metabolites in plasma after i.v. application of [13C]-Cy-3-glc. Some metabolites were identified 15 s after administration [28].

In the present study approximately 78 mg (167 µmol) DG and 9.7 mg (16.7 µmol) CS were consumed resulting in a nanomolar concentration range. The blood concentrations measured for the two key parameters are in line with the concentrations found in plasma following consumption of berries or grapes in human studies [12]. In a study by Wu et al. maximum plasma levels for total anthocyanins were reported with 1–100 nmol/L [29]. 

In the literature, anthocyanins were found in the human blood within a few minutes [25], which is also noted in the present study as some subjects had their individual T_max_ values after 0.5 h. This fast increase of anthocyanins may be explained by the start of uptake already in stomach. The resorption continues in the small intestines and to some extent possibly also in the colon. Anthocyanins are quite prone to metabolism (phase I and phase II metabolites, conjugate products, microbe-generated metabolites etc.). The relative contributions of each of these classes of metabolites to human health maintenance are still under investigation [24]. Nevertheless, there is also evolving evidence of specific molecular targets by which anthocyanins might also address their health benefits. In this context, it is of great interest to characterize the uptake of unmetabolized anthocyanin structures present in the extract. This also allows a better understanding of the transfer of data found in in vitro experiments in which extracts are directly investigated without prior physiologic metabolism. The data presented clearly show that from anthocyanin structures present in the MBE there is an appreciable amount taken up in its unmetabolized amounts, which was demonstrated for DG and CS. 

### 4.2. Gallic Acid (GA) and Protocatechuic Acid (PCA)

GA and PCA are phenolic acids with abundant occurrence in fruits but also known as breakdown products of anthocyanins and other polyphenols. Within the study, the phenolic acids GA and PCA were additionally determined in the blood samples to estimate extent of metabolism activity. Plasma levels for both acids were observed in very low range. Nevertheless, plasma levels increased after intake of MBE compared to baseline. GA is the breakdown product of delphinidin [25], whereas PCA is the main human breakdown product of cyanidin compounds [30]. The metabolism might occur throughout the entire gastrointestinal tract including microbiome, as well as during systemic metabolism. For interpretation of the data, one has to keep in mind that the study product was provided on an empty stomach and thus a very fast transit is expected. 

From the data presented, the slight increase was very early with on average 0.8 ± 0.3 h for PCA and 1.6 ± 1.5 h for GA. In comparison, Vitaglione et al. [30] described the maximum serum concentration of PCA at 2 h after consumption of 1 L of blood orange juice with 71 mg cyanidin-glucosides. Pimpao et al. also reported very low concentrations of GA after intake of anthocyanins provided as mixed berry fruit puree [31]. Although GA was detectable in all volunteers in the Pimpao study [31], it was mostly found under the limit of quantification; only being quantifiable in three out of nine volunteers, reaching a maximum concentration at 1 h after ingestion of the fruit puree despite the puree itself contained remarkable concentrations of GA with 425.9 mg/500 mL. A reason for these low concentrations might be that the phenolic acid is further degraded [25]. Anyhow, the increase of both breakdown products as a consequence of the intake of MBE could clearly be demonstrated within the study. 

### 4.3. Reporting Limitations

The data present the uptake of MBE under standardized conditions which was well tolerated and no adverse events were reported. No statements can be made on other factors influencing bioavailability, for example, intake together with other food components like proteins. The nutrition during the kinetic day was chosen with regard to a minimal amount of polyphenol intake. Existing literature shows that certain dietary components can modify anthocyanin absorption [32,33]. Furthermore, the presence of other flavonoids could possibly interfere with the absorption of anthocyanins [34]. To minimize the impact of anthocyanins/flavonoids present in fruits and vegetables, subjects were advised to consume an anthocyanin and polyphenol low diet three days prior to the kinetic day. The compliance to these restrictions was confirmed as baseline levels of the selected key substances were not detectable.

The standardized meals were served 2 h and 5 h after intake of the test product. As the maximum of DG and CS were already observed prior to the served meals, no impact of the foods provided during the kinetic day is expected. 

## 5. Conclusions

The results confirmed a fast uptake and metabolism of the two selected key substances. The profile slightly differed between DG and CS with a later occurring peak for CS. The phenolic acids GA and PCA as breakdown products of anthocyanins were observed and confirmed, although concentrations were below the limit of quantification. 

Taken together the present study clearly demonstrates the bioavailability and thus the uptake of unmetabolized, native anthocyanins present in the proprietary MBE. In further research, the mode of action and identification of biologically active anthocyanins responsible for the observed health benefits should be investigated. 

## Figures and Tables

**Table 1:** Demographic and baseline data

Variable	Mean	95% CI
Age (years)	29.5	(23.4; 35.7)
BMI (kg/m^2^)	23.6	(21.6; 25.7)
Systolic BP	122.5	(111.7; 133.3)
Diastolic BP	74.7	(70.4; 79.0)
Fruit consumption/day	1.5	(1.1; 1.8)
Vegetable consumption	1.7	(1.2; 2.2)
Haemoglobin	13.6	(12.9; 14.3)
Total Cholesterol	182.8	(160.4; 205.2)
GOT	23.2	(14.7; 31.6)

## Supplementary Materials

The following are available online at http://www.mdpi.com/2072-6643/10/11/1720/s1, Figure S1: Chromatogram proprietary Maqui Berry Extract, Figure S2: MRM channels of the standards delphinidin-3-*O*-glucoside (DG) (a) and cyanidin-3-*O*-sambubioside (CS) (b) and internal standard in spiked plasma sample, Figure S3. Example MRM for (a) DG, (b) CS, (c) IS, (d) GA and (e) PCA, Table S1: Gradient UPLC-Separation, Table S2: MRM transitions and instrument settings.
