# Supplementary material for: Bioavailability Study of Maqui Berry Extract in Healthy Subjects
Source: Nutrients. 2018 Nov 9;10(11):1720. doi: 10.3390/nu10111720 (PMC6267473; doi:10.3390/nu10111720)
Supplement: Supplementary file 1 [file nutrients-10-01720-s001.pdf]

Supplementary material

Figure S1: Chromatogram proprietary Maqui Berry Extract

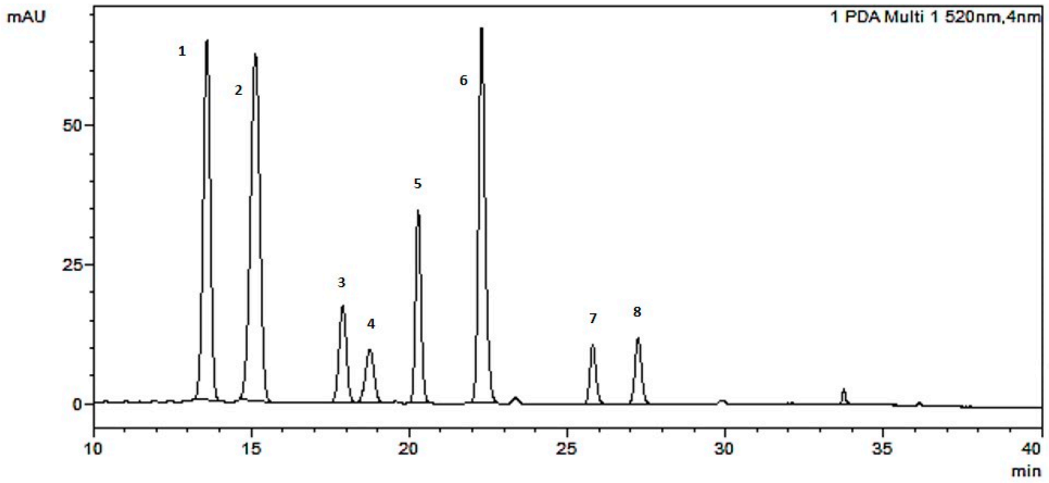

ID #	Name	Area
1	Delphinidin-3-*O*-sambubioside-5-o-glucoside	1013361
2	Delphinidin-3,5-*O*-diglucoside	1287946
3	Cyanidin-3-*O*-sambubiodise-5-*O*-glucoside	279426
4	Cyanidin-3,5-diglucoside	176784
5	Delphinidin-3-*O*-sambubiodise	420646
6	Delphinidin-3-*O*-glucoside	976347
7	Cyanidin-3-*O*-sambubioside	139714
8	Cyanidin-3-*O*-glucoside	166323

Table S1 : Gradient UPLC-Separation
Buffer A: 5 % formic acid in water
Buffer B: acetonitrile

Gradient	Time	Flow (mL/min)	%A	%B
1	initial	0.7	99.0	1.0
2	0.50	0.7	99.0	1.0
3	1.00	0.7	95.0	5.0
4	6.00	0.7	75.0	25.0
5	6.10	0.7	40.0	60.0
6	7.00	0.7	99.0	1.0
7	8.00	0.7	99.0	1.0

Table S2: MRM transitions and instrument settings

Mode	Analyte	Retention Time[min]	Precursor Ion [m/z]	Daughter Ion [m/z]	MRM Class	Dwell Time [s]	Cone Voltage [V]	Collision Energy [V]
ESI+	DG	2.69	465.2629	303.1602	Quantifier	0.018	52	18
ESI+	DG	2.69	465.2629	229.1036	Qualifier	0.018	52	54
ESI+	CS	3.03	581.3102	287.1071	Quantifier	0.018	54	22
ESI+	CS	3.03	581.3102	137.0949	Qualifier	0.018	54	70
ESI-	IS	4.13	246.0148	199.9748	Quantifier	0.018	12	4
ESI-	IS	4.13	200.0148	155.9845	Qualifier	0.018	30	8
ESI-	PCA	0.68	152.9947	108.9023	Quantifier	0.025	40	12
ESI-	PCA	0.68	199.0585	153.0067	Qualifier	0.025	8	6
ESI+	GA	0.33	171.1173	127.0570	Quantifier	0.025	20	10
ESI+	GA	0.33	171.1173	153.0844	Qualifier	0.025	20	10

Figure S2: MRM channels of the standards delphinidin-3-*O*-glucoside (DG) (a) and cyanidin-3-*O*-sambubioside (CS) (b) and internal standard in spiked plasma sample

(a)

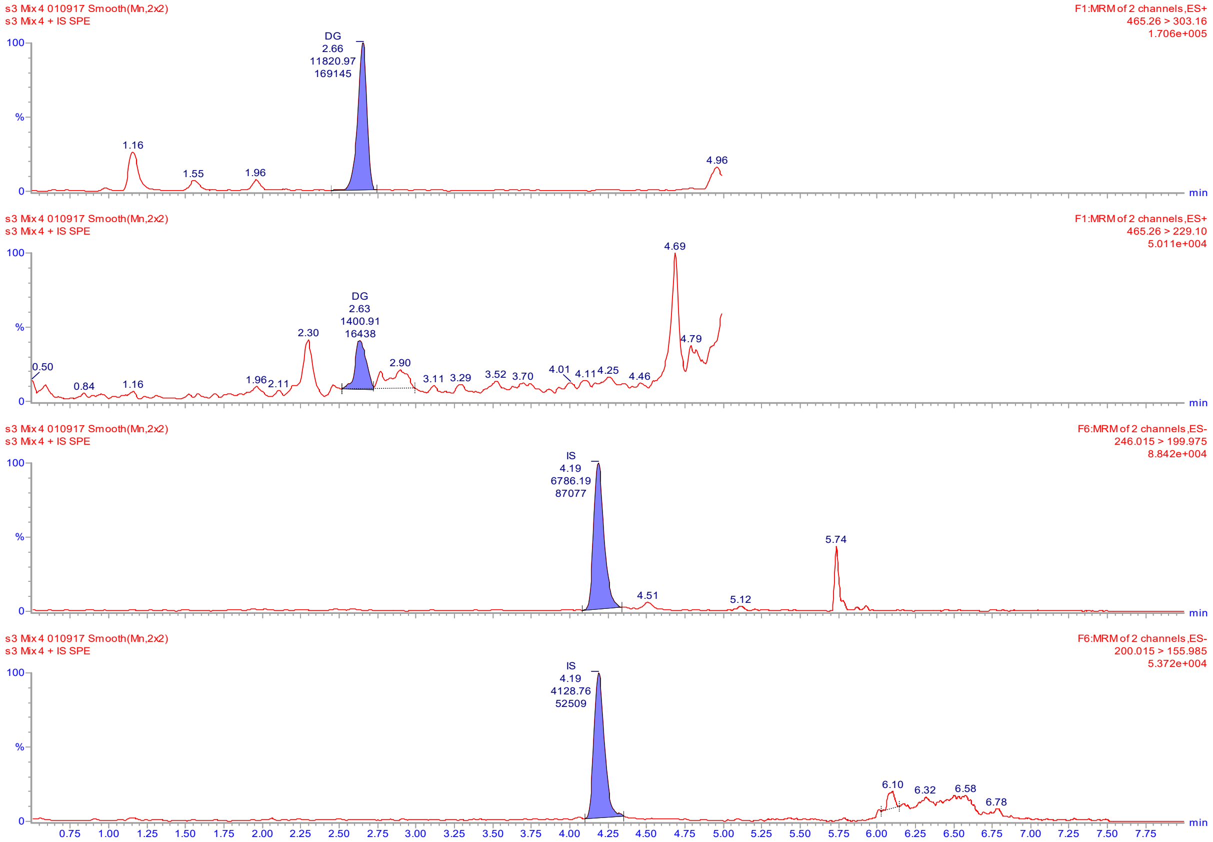

(b)

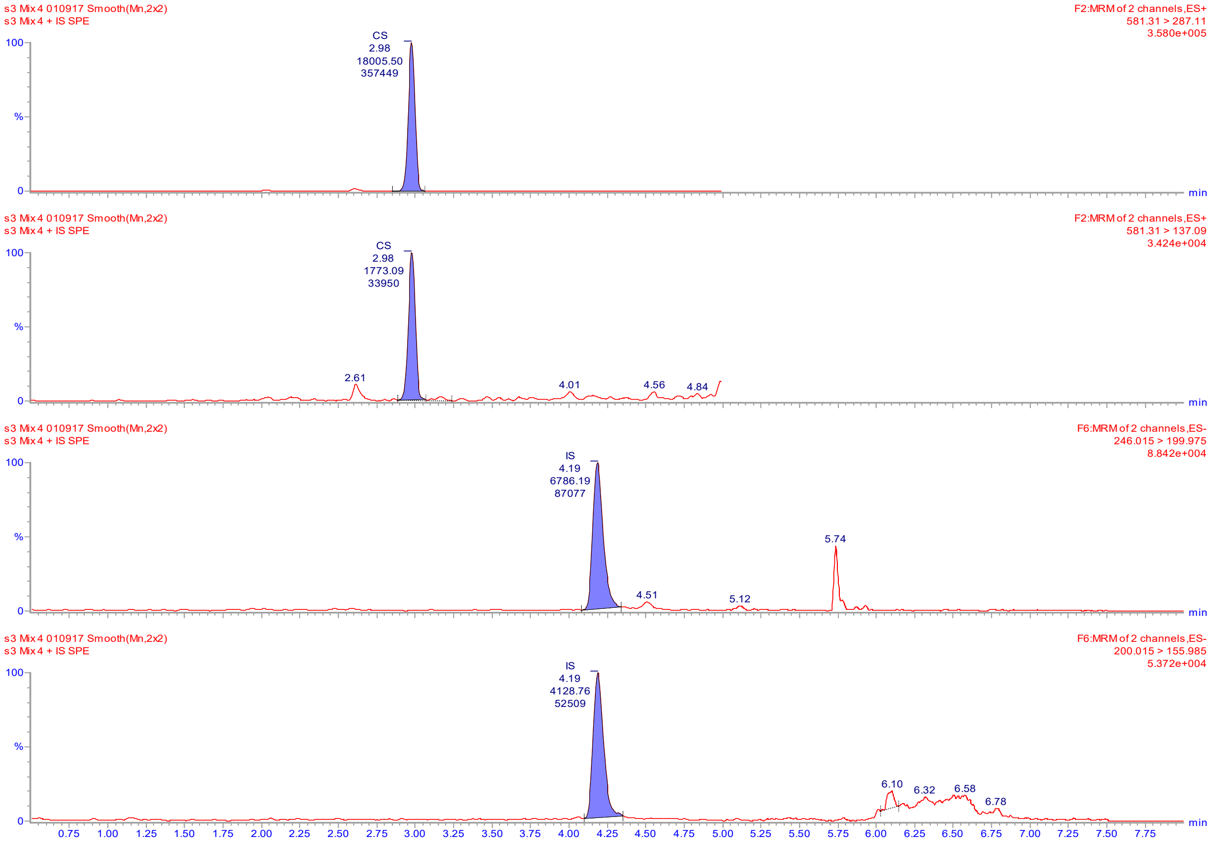
s3 Mix 4 010917 Smooth(Mn,2x2)
s3 Mix 4 + IS SPE
F2:MRM of 2 channels,ES+
581.31 > 287.11
3.580e+005
CS
2.98
18005.50
357449
s3 Mix 4 010917 Smooth(Mn,2x2)
s3 Mix 4 + IS SPE
F2:MRM of 2 channels,ES+
581.31 > 137.09
3.424e+004
CS
2.98
1773.09
33950
2.61
4.01
4.56
4.84
s3 Mix 4 010917 Smooth(Mn,2x2)
s3 Mix 4 + IS SPE
F6:MRM of 2 channels,ES-
246.015 > 199.975
8.842e+004
IS
4.19
6786.19
87077
4.51
5.12
5.74
s3 Mix 4 010917 Smooth(Mn,2x2)
s3 Mix 4 + IS SPE
F6:MRM of 2 channels,ES-
200.015 > 155.985
5.372e+004
IS
4.19
4128.76
52509
6.10
6.32
6.58
6.78
100
%
0
min
0.75 1.00 1.25 1.50 1.75 2.00 2.25 2.50 2.75 3.00 3.25 3.50 3.75 4.00 4.25 4.50 4.75 5.00 5.25 5.50 5.75 6.00 6.25 6.50 6.75 7.00 7.25 7.50 7.75

Figure S3. Example MRM for (a) DG, (b) CS, (c) IS, (d) GA and (e) PCA

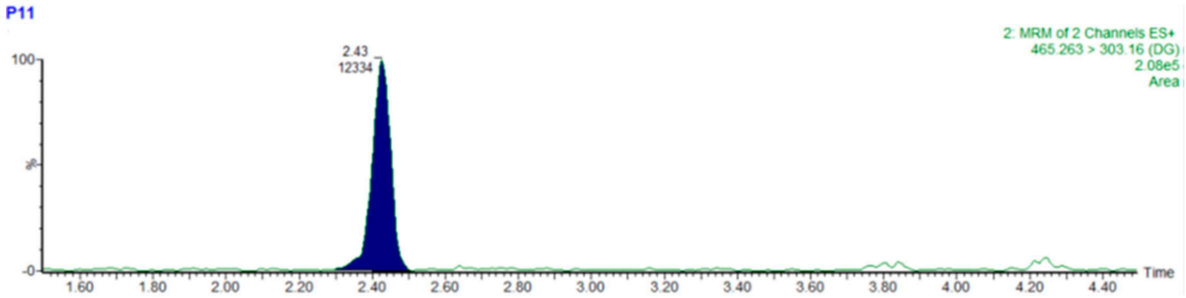

(**a**)

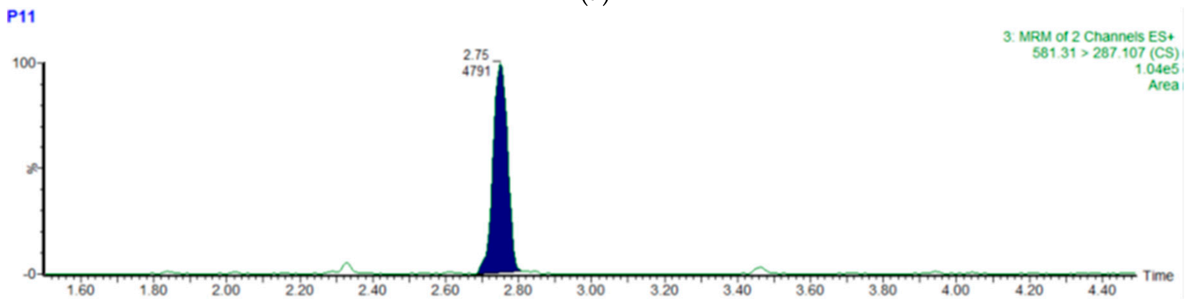

(**b**)

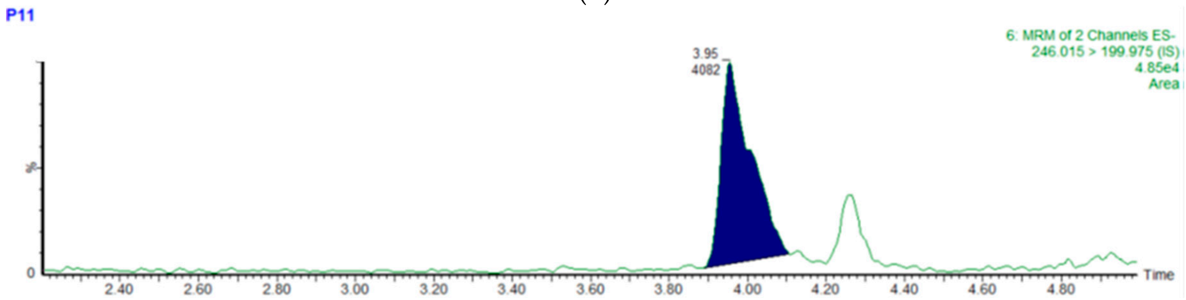

(**c**)

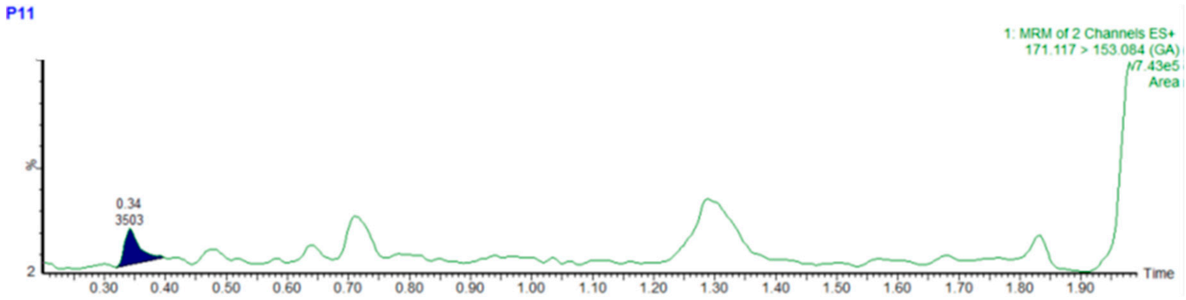

(**d**)

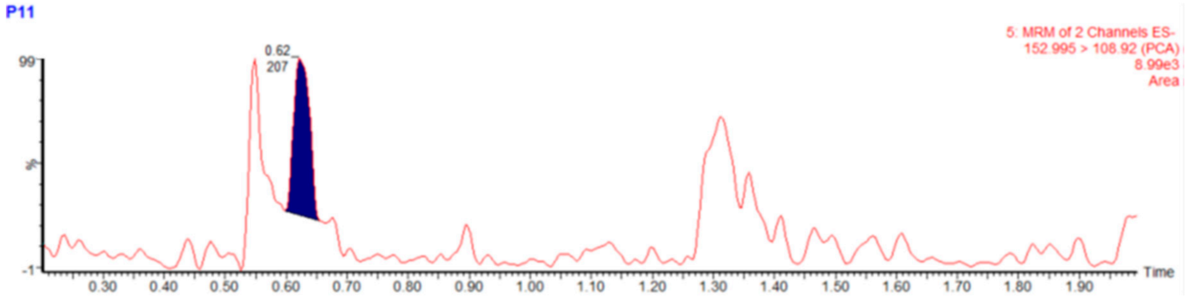

(**e**)
